# Supplementary material for: A Novel Noninvasive Technique for Intracranial Pressure Waveform Monitoring in Critical Care
Source: J Pers Med. 2021 Dec 5;11(12):1302. doi: 10.3390/jpm11121302 (PMC8707681; doi:10.3390/jpm11121302)
Supplement: Supplementary file 1 [file jpm-11-01302-s001.zip › jpm-1430641-supplementary.pdf]

A NOVEL NONINVASIVE TECHNIQUE FOR INTRACRANIAL COMPLIANCE MONITORING IN NEUROCRITICAL CARE

*Corresponding author:
Sérgio Brasil, MD, PhD
Division of Neurosurgery - University of São Paulo
255 Enéas Aguiar Street
Sao Paulo
Zipcode05403000
sbrasil@alumni.usp.br

Supplemental Table S1. STARD 2015 criteria for the study structure.

Section & Topi	No	Item	page #
**TITLE**			
	**1**	Identification as a study of diagnostic accuracy using at least one measure of accuracy (such as correlation, sensitivity, specificity, predictive values, or AUC)	1
**ABSTRACT**			
	**2**	Structured summary of study design, methods, results, and conclusions (for specific guidance, see STARD for Abstracts)	2
**INTRO**			
	**3**	Scientific and clinical background, including the intended use and clinical role of the index test	3
	**4**	Study objectives and hypotheses	4
**METHODS**			
Study design	**5**	Whether data collection was planned before the index test and reference standard were performed (prospective study) or after (retrospective study)	4
Participants	**6**	Eligibility criteria	4
	**7**	On what basis potentially eligible participants were identified (such as symptoms, results from previous tests, inclusion in registry)	4

	**8**	Where and when potentially eligible participants were identified (setting, location and dates)	5
	**9**	Whether participants formed a consecutive, random or convenience series	5
Test methods	**10a**	Index test, in sufficient detail to allow replication	7
	**10b**	Reference standard, in sufficient detail to allow replication	6
	**11**	Rationale for choosing the reference standard (if alternatives exist)	6
	**12a**	Definition of and rationale for test positivity cut-offs or result categories of the index test, distinguishing pre-specified from exploratory	6
	**12b**	Definition of and rationale for test positivity cut-offs or result categories of the reference standard, distinguishing pre-specified from exploratory	6
	**13a**	Whether clinical information and reference standard results were available to the performers/readers of the index test	6
	**13b**	Whether clinical information and index test results were available to the assessors of the reference standard	6
